# Influence of Geographic Origin and Plant Source on Physicochemical Properties, Mineral Content, and Antioxidant and Antibacterial Activities of Moroccan Propolis

**DOI:** 10.1155/2021/5570224

**Published:** 2021-03-19

**Authors:** Naoual El Menyiy, Meryem Bakour, Asmae El Ghouizi, Soukaina El Guendouz, Badiaa Lyoussi

**Affiliations:** Laboratory of Natural Substances, Pharmacology, Environment, Modeling, Health, and Quality of Life (SNAMOPEQ), Faculty of Sciences Dhar Mehraz, Sidi Mohamed Ben Abdellah University, Fez, Morocco

## Abstract

This research is aimed at determining the physicochemical properties (resin, wax, balsams, pH, moisture, ash, and mineral contents) of propolis samples collected from different geographical areas in Morocco, as well as evaluating the antioxidant and antibacterial activities of these samples. The results showed the following values for physicochemical analysis: resin (17.42-58.01%), wax (21.31-70.12%), balsam (0.27-2.12%), pH (3.7-5.3), moisture (1.02-3.65%), and ash (0.72-5.01%). The phenolic and flavone/flavonol contents of samples were ranged between 6.74 mg FAE/g and 149.13 mg FAE/g and between 1.19 mg QE/g and 108.11 mg QE/g, respectively. The sample P3 presented also the strongest radical scavenging activity toward DPPH, ABTS free radicals, and FRAP assay with IC_50_ values of 0.021, 0.026, and 0.042 mg/mL, respectively. All propolis samples showed significant inhibitory effects against all tested microorganisms with MICs ranging from 0.28 mg/mL to 1.12 mg/mL for Gram-negative strains and from 0.002 mg/mL to 1.12 mg/mL for Gram-positive strains. A strong correlation was found between resin, total phenolic compounds, flavones/flavonols, and antioxidant activity. Linear discriminant analysis revealed that the samples studied were divided into two groups which were differentiated by the data of antioxidant activity, mineral contents, and antibacterial activity. It can be concluded that the physicochemical properties, mineral content, and biological activities of Moroccan propolis depend on their geographical and botanical origin.

## 1. Introduction

Propolis or “bee glue” is a resinous substance collected by honeybees (*Apis mellifera* L.) from the resin found on the buds of trees and plants [1]. This material is transported to the hive and mixed with 13-glycosidase enzymes of their saliva, partially digested, and added to bee wax and pollen producing a strongly adhesive substance, which is used by bees for the construction and maintenance of hives [2, 3]. In general, this bee product contains 50% resins, 30% waxes, 10% essential oils, 5% pollen, and 5% other organic compounds, including the phenolic compounds, amino acids, esters, flavonoids, and terpenes [4].

Propolis is used for therapeutic purposes since ancient times [5]. Nowadays, several studies shed light on the biological activities of propolis, such as antimicrobial [6, 7], anti-inflammatory [8], antiviral [9], hepatoprotective [10], immunomodulatory [11], antioxidant [12, 13], and anticancer activities [14].

A considerable amount of literature has been published on the biological activities of Moroccan propolis. El Menyiy et al. reported that Moroccan propolis has a potential to prevent urinary against calculus, crystalluria, and proteinuria [15]. Similarly, other studies showed that Moroccan propolis has a protective effect against renal, hepatic, and hematological toxicity caused by paracetamol and chromium [16, 17].

Recently, *in vivo* studies have shown that Moroccan propolis exhibited a promising antidiabetic activity; it significantly decreased blood glucose level, increased insulin sensitivity and pancreatic *β* cell function, ameliorated dyslipidemia by the decrease of TC, TG, LDL-C, and VLDL and the increase of HDL, and prevented diabetic complications such as liver and kidney injury [18, 19]. Other reports showed that Moroccan propolis has a strong antioxidant, antibacterial, anti-inflammatory, and anticancer potential [20–24]. Importantly, the chemical composition of Moroccan propolis revealed the presence of caffeic acid, p-coumaric acid, ferulic acid, naringenin, pinocembrin, chrysin, galangin, pinobanksin, and quercetin, which are known for their broad spectrum of biological activity [11, 21].

The biological effects and chemical composition of propolis vary according to many factors such as geographical origin, botanical source, honey bee species, climate environmental conditions, and collecting season [25, 26]. Therefore, the main objective of the present work was to investigate the physicochemical parameters and antioxidant and antibacterial activities of twenty propolis samples collected from different botanical sources and geographical origins in Morocco.

## 2. Materials and Methods

### 2.1. Propolis Extraction

Twenty samples of propolis were collected from different regions of Morocco by professional beekeepers. The cities of sampling and the predominant vegetation in each city are presented in Table 1. The samples were extracted by maceration using the method described by El-Guendouz et al. [23] with some modifications. One gram of each propolis sample was macerated in 10 mL of 70% ethanol for 7 days at room temperature under physical agitation. The final extract was filtered and centrifuged at 4,000 rpm for 10 minutes, and the supernatant was used for successive analyses.

### 2.2. Physicochemical Characterization of Propolis Samples

#### 2.2.1. pH

The pH meter was used to determine the pH value of propolis solution that was prepared by dissolving five grams of each propolis sample in 30 mL of methanol [27].

#### 2.2.2. Ash Content

For the determination of the ash content, one gram of each sample was placed in a crucible in a muffle furnace and heated at 550°C for five hours. The results were expressed as percentage *w*/*w* [28].

#### 2.2.3. Moisture Content

The AOAC method was used to determine the moisture content of propolis samples [29]. Briefly, one gram of propolis sample was placed in a crucible in a furnace and heated at 105°C to a constant weight.

#### 2.2.4. Wax, Resin, and Balsam Contents

The content of wax, resin, and balsam in different propolis samples was estimated according to the methods described by Papotti et al. [30], with slight modification. Briefly, one gram of propolis was macerated with 40 mL of petroleum ether at 40−60°C under stirring for 48 h. The extract was added to 40 mL of 70% ethanol, heated under reflux until a clear solution was obtained, and then cooled at 0°C for 1 h to promote wax separation. The results were expressed as a percentage (*w*/*w*), representing the rate of wax in each propolis sample. Concerning the resin content, the residual propolis obtained after the wax extraction was macerated with 40 mL of chloroform and ethanol 1 : 1 (*v*/*v*) under stirring for 48 h. The extract was concentrated in a rotary evaporator to obtain a solid residue; the results were expressed as % *w*/*w*. The balsam content was estimated as follows: the 70% ethanolic filtrate obtained during the wax extraction was concentrated under reduced pressure at 60°C. Then, 10 mL of dichloromethane was added to the aqueous residue; the organic phase was collected and dried over 6 g of anhydrous Na_2_SO_4_ and then filtered. The solution was evaporated to dryness under reduced pressure at 60°C. The results were expressed as a percentage (*w*/*w*), representing the rate of balsam in each propolis sample.

### 2.3. Mineral Content

The mineral content of propolis samples was determined by Inductively Coupled Plasma Mass Spectrometry (ICP-MS) following the procedure described by Silva et al. [31]. Briefly, 5 mL of 0.1 M nitric acid was added to the ashes and heated to complete dryness. Then, 10 mL of 0.1 M nitric acid was added, and the volume was made up to 25 mL with distilled water. The values were calculated as mg of each mineral element per kg of propolis.

### 2.4. Antioxidant Properties of Propolis Samples

#### 2.4.1. Total Phenolic Content

Folin-Ciocalteu's method was used to determine the polyphenol content in propolis samples according to the procedure described by El-Guendouz et al. [23]. The resulting values were expressed as mg ferulic acid equivalents per g of propolis (mg FAE/g).

#### 2.4.2. Flavone and Flavonol Content

The aluminium chloride was used to determine the flavone and flavonol content in propolis samples according to the method described by El-Guendouz et al. [23]. The resulting values were expressed as mg quercetin equivalents per g of propolis (mg QE/g).

### 2.5. Antioxidant Activity

#### 2.5.1. Total Antioxidant Capacity

The phosphomolybdenum method was used to evaluate the total antioxidant capacity of the propolis extract as described by Zengin et al. [32]. The resulting values were expressed as milligram equivalents ascorbic acid per gram of propolis (mg AAE/g propolis).

#### 2.5.2. Free-Radical Scavenging Activity (DPPH)

The ability of propolis extract to scavenge the radical 2,2-diphenyl-1-picrylhydrazyl (DPPH) was evaluated using the method of Miguel et al. [33]. The absorbance was recorded at 517 nm, the values of IC_50_ were determined, and BHT was used as positive control.

#### 2.5.3. Scavenging Activity of ABTS Radical Cation

The ability of propolis extract to scavenge ABTS radical was monitored using the procedure of Miguel et al. [33]. The absorbance was read at 734 nm, and the values of IC_50_ were determined. Gallic acid was used as positive control.

#### 2.5.4. Reducing Power Determination

The reductive potential of propolis extract was evaluated following the procedure of Moreira et al. [34]. The absorbance was recorded at 700 nm, and IC_50_ was determined. The test has been done in triplicate, and ascorbic acid was used as positive control.

### 2.6. Antibacterial Activity of Propolis Samples

#### 2.6.1. Bacterial Strains

The antibacterial activity of propolis extracts was evaluated against four bacterial strains: two Gram-negative strains (*Escherichia coli* BLSE (ATB:87) and *Pseudomonas aeruginosa*) and two Gram-positive strains (*Streptococcus faecalis* and *Staphylococcus aureus*); bacterial strains were obtained from the Hassan II University Hospital and Laboratory of Microbiology, Faculty of Medicine and Pharmacy Fez.

#### 2.6.2. Disk Diffusion Method

The disk diffusion method was used to evaluate the antibacterial activity of hydroethanolic extract of propolis [35]. About 20 mL of Mueller-Hinton agar (MHA) medium was poured into Petri plates. Each Petri plate was inoculated with a bacterial inoculum consisting of 0.5 McFarland (1‐2)∗10^8^ CFU/mL which was prepared in a physiologic saline buffer. Then, sterile Whatman paper disks were placed on a medium and impregnated with 10 *μ*L of hydroethanolic extracts of propolis (100 mg/mL). Ethanol 70% was used as a negative control, to check the possible activity of the solvent of extraction against the tested bacterial strains. After 24 h incubation at 37°C, the inhibition zone was measured in mm. Each experiment was carried out in triplicate.

#### 2.6.3. Minimum Inhibitory Concentration (MIC)

The MIC of each extract was determined following the NCCLS method [36]. It was performed by a serial dilution of propolis extracts which was made in a concentration ranged between 0.048 and 100 mg/mL. 10 *μ*L of each concentration was mixed in a 96-well plate with 180 *μ*L of MH broth and 10 *μ*L of bacterial inoculums (5 × 10^5^ CFU/mL). The concentration of ethanol in each well does not exceed 3.5% and was used as a negative control. After 20 h incubation of the microplates at 37°C, 20 *μ*L of 2,3,5-triphenyl tetrazolium chloride (TTC) was added to each well and incubated for 30 min. MIC corresponds to the lowest concentration of the extract that inhibited visible growth (indicated by the absence of red colour after the adding of TTC) [37].

#### 2.6.4. Minimal Bactericidal Concentration (MBC)

In Muller-Hinton agar (MHA), each well which the concentration is ≥MIC was subcultured and incubated at 37°C for 24 h. MBC corresponds to the lowest concentration of the extracts that killed 99.9% from the inoculated bacteria. The antibacterial effect was considered bactericidal if MBC/MIC = 1‐2 and bacteriostatic if MBC/MIC = 4 to 16 [38].

### 2.7. Statistical Analysis

One-way ANOVA followed by post hoc Tukey's multiple comparison test using GraphPad Prism 5 software was used for statistical comparisons. Pearson correlation coefficient (*r*) was used to analyze the correlations between different parameters of propolis samples. The results were also subjected to linear discriminant analysis (LDA) using program PAST: paleontological statistics software package for education and data analysis, version 3.20.

## 3. Results and Discussion

### 3.1. Physicochemical Characterization

The resin and wax are the main compounds in propolis with a rate of 50% and 30%, respectively [39]. In the present work, all the physicochemical results of twenty propolis samples are summarized in Table 2; the analysis of the results indicates that the samples have varying amounts of resin, wax, and balsam with significant differences between them. Propolis samples with high wax content had low resin content. As indicated in Table 2, the high wax content is presented in sample P20 and the lowest content in sample P3. For the balsams, sample P6 showed the high content, while the lowest content was obtained in sample P12. The other samples have intermediate balsam content ranged between 0.29 ± 0.01% and 1.89 ± 0.01%. Concerning the resin contents, sample P3 shows the highest value, followed by sample P7. The highest content in the wax, balsam, and resin found in Moroccan propolis samples in this study is in agreement with the range of those generally detected in Italian propolis samples in which resin content ranged between 39.1 and 72.7% while wax content ranged between 12.8 and 41.0% [40]. Determination of ash content is an indication that can identify a possible adulteration in propolis samples [41]. The ash content values of all investigated samples ranged from 0.72 ± 0.02% to 5.01 ± 0.01%. All samples agree with the limit established by the Brazilian legislation [42]. Moreover, moisture is also an indication of the quality of propolis; the high water content in propolis indicates inadequate storage and manipulation conditions [43]. The results obtained showed also that moisture content in all analyzed samples does not exceed 3.65 ± 0.01%; this value is within the limit established by the Brazilian legislation (not more than 8%) [42]. The pH results of the samples were all somewhat acidic ranging between 3.7 ± 0.2 in sample P20 to 5.3 ± 0.12 in samples P3, P7, and P16. The pH values of all investigated samples are in agreement with those obtained in other studies [27, 44, 45].

### 3.2. Mineral Content

Table 3 shows the results obtained for the mineral content of twenty Moroccan propolis samples. The concentration of all macro- and microelements was widely varied; the calcium content was the most dominant minerals in all investigated samples and ranged between 210 ± 31.1 mg/kg in sample P20 and 1325 ± 16.1 mg/kg in sample P3. The sodium content was the second dominant minerals ranged between 51 ± 10.16 mg/kg and 690 ± 13.11 mg/kg, followed by potassium content ranging between 116 ± 21.15 mg/kg and 705 ± 25.28 mg/kg, followed by magnesium content which varies from a minimum value of 58 ± 21.1 mg/kg to a maximum of 950 ± 11.17 mg/kg, while the toxic elements Cd, Cr, Co, and Ni are not detected in all samples. However, the Pb was detected in samples P13 (0.027 ± 0.02 mg/kg) and P20 (0.02 ± 0.01 mg/kg).

It has been shown that the variation of the mineral elements in the propolis depends on several factors such as the mineral composition of the source plants, humidity, and soil pH [43]. Other studies showed that the amount of toxic elements depends on the method of harvesting propolis [46]. The profile in mineral elements differs from one sample to another even for samples from the same region of Morocco; therefore, it can be a parameter determining the geographical and botanical origins of propolis [47]. Similar results were reported previously in other studies [43, 47, 48].

### 3.3. Bioactive Compounds and Antioxidant Activity

Propolis is constituted mainly by flavonoids, hydroxybenzoic acids, hydroxycinnamic acids, and stilbenes that contribute to their functional properties, including antioxidant and antimicrobial [49, 50].  Table 4 shows the quantitative difference for total phenolic, flavones, and flavonols in propolis from the different areas of Morocco. For the total phenolic compounds, the concentrations ranged between 6.74 ± 1.17 mg FAE/g in sample P20 and 149.13 ± 2.12 mg FAE/g in sample P3. The same observation was made for flavones and flavonols where the highest concentration was found in sample P3 (108.11 ± 0.51 mg QE/g), immediately followed by samples P7 (46.51 ± 3.08 mg QE/g) and P2 (40.90 ± 1.42 mg QE/g). The concentrations of total phenolic compounds, flavones, and flavonols found in our propolis samples were within the range obtained in Portuguese, Algerian, and Chinese propolis [51–53].

On the other hand, it is well known that propolis exhibits a strong antioxidant activity [2, 54]. The results of the present work showed a considerable antioxidant activity in all propolis where the sample from Outat el Haj presented the high total antioxidant capacity (80.82 ± 2.16 mg AAE/g) and the best antioxidant activity in the three tests DPPH, ABTS, and reducing power assay, with IC_50_ values of 0.021 ± 0.001 mg/mL, 0.026 ± 0.0007, and 0.042 ± 0.001 mg/mL, respectively, while the sample from Tantan presented the lowest activity with IC_50_ values of 1.308 ± 0.018, 1.529 ± 0.015, and 1.512 ± 0.106 mg/mL in DPPH, ABTS, and reducing power assay, respectively. A significant negative correlation between IC_50_ values and total phenols, resin, flavones, and flavonols (*p* < 0.01) was found (Table 5). It was observed that propolis with high resin, phenolic, flavone, and flavonol contents has the highest antioxidant activity, which means that the antioxidant activity of the propolis sample may be due to their richness in these groups of compounds. This correlation has also been revealed for Moroccan propolis in several studies [20, 21, 33].

In sum, the screening of different Moroccan propolis samples from various regions, regarding their antioxidant activities, revealed a significant difference among them. As mentioned, the observed differences could be related to the compounds exhibiting the antioxidant capacity in each propolis sample, which, in turn, depends on the flora and geographic origin [55, 56].

### 3.4. Antibacterial Activity

The antibacterial activity for propolis is one of the most documented biological properties in the literature [57, 58].  Table 6 summarizes the results of the antibacterial activity of hydroethanolic extracts of propolis. All propolis samples presented high antibacterial effects, mostly against Gram-positive bacterial strains.

The antibacterial activity of propolis using the disk diffusion method showed an inhibition diameter ranged between 12.3 ± 1.5 and 32.2 ± 1.1 mm for *Staphylococcus aureus* followed by *Streptococcus faecalis*, with a diameter ranged from 10 ± 0.12 to 31.5 ± 1.1 mm, and *Escherichia coli* with a diameter varied from 8.12 ± 1.09 to 19.33 ± 2.51 mm. The *Pseudomonas aeruginosa* has the lowest value of the inhibition diameter (8.02 ± 0.5 to 12.3 ± 0.5 mm). The propolis sample P3 which has high resin, phenol, flavone, and flavonol contents and higher total antioxidant was the most effective against all bacteria.

Concerning the minimum inhibitory concentrations and the minimum inhibitory concentrations, for all strains, the MIC and MBC values varied between 0.002 ± 0.0001 mg/mL and 1.12 ± 0.01 mg/mL, respectively. Among, the bacterial strains tested, *Staphylococcus aureus* was the most susceptible as compared to other bacteria, while *Streptococcus faecalis* was the most resistant. These results have also been confirmed by several studies demonstrating the strong antimicrobial activity of propolis extract against bacteria especially Gram-positive strains [59–61]. Propolis sample from Outat el Haj presented the best antibacterial effect with the lowest value of MIC (0.28 ± 0.02, 0.002 ± 0.0001, 0.56 ± 0.01, and 0.07 ± 0.001 mg/mL) for *Escherichia coli*, *Staphylococcus aureus*, *Pseudomonas aeruginosa*, and *Streptococcus faecalis*, respectively, followed by the sample from Salé with MIC values of 0.28 ± 0.02, 0.002 ± 0.0001, 0.56 ± 0.01, and 0.28 ± 0.01 mg/mL for *Escherichia coli*, *Staphylococcus aureus*, *Pseudomonas aeruginosa*, and *Streptococcus faecalis*, respectively. However, propolis sample from Tantan and ethanol have no effect against all bacteria tested.

The antibacterial effect of propolis could be attributed to its phenolic compounds. It is well known that phenolic acids and flavonoids nullify the potential of the internal bacterial membrane, decrease the production of ATP, and inhibit DNA gyrase involved in the synthesis of bacterial DNA and RNA which leads to the inhibition of bacterial viability [62, 63]. Interestingly, numerous researchers improved the toxic effects of minerals such as iron, phosphorus, cooper, and zinc on both Gram-positive and Gram-negative bacteria [58, 64, 65]; therefore, the richness in macro- and microelements gives propolis an additional potential antibacterial.

The difference observed in the antibacterial activity of the propolis samples could be attributed to the diversity of the bioactive molecules presented in each sample which is related to the botanical and geographical origin of propolis and climatic conditions [66–68].

### 3.5. Linear Discriminant Analysis (LDA)

**Figure 1:**
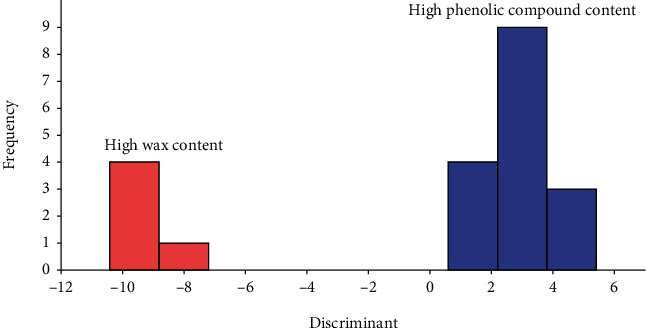
Histogram of canonical discriminant function considering the content of wax and phenolic compounds in propolis samples.

Figure 1 shows the results of linear discriminant analysis addressing each sample of propolis to one of the following groups: group 1 composed by samples with high wax content (>30%) [69] and group 2 composed by samples with high content of phenolic compounds using antioxidant activity data, data of minerals present and data of antibacterial activity as dependent variables. The analysis allowed us to obtain just one canonical function that clearly separates the two groups. All propolis samples were correctly classified by LDA (100%); these results showed that the two groups of propolis can be differentiated by considering the mentioned variables. Similar results were observed in a study concerning seven Moroccan propolis, which confirmed that samples with high wax content have low phenolic compounds and low antimicrobial activities [21].

## 4. Conclusion

Overall, it can be concluded that Moroccan propolis with low wax content has high content of antioxidant compounds and high mineral contents and exhibits important biological activities. These findings could be related to the geographical and botanical origin of propolis, besides the good beekeeping practices and the choice of the right propolis traps to harvest clean and good quality propolis.

The present study will be helpful for the standardization of Moroccan propolis and could provide useful information for food and nutraceutical industries to choose high-quality propolis. Therefore, a thorough chemical characterization of a large number of samples is necessary to cover all aspects of propolis quality.

## Figures and Tables

**Table 1:** Predominant vegetation in the geographical locations of the propolis samples.

Propolis	City	Predominant vegetation
P1	Séfrou	*Bupleurum*, *Ceratonia*, and *Eucalyptus*
P2	Moulay Yaâcoub	*Ceratonia*, *Citrus*, *Eucalyptus*, and *Silybum*
P3	Outat el Haj	*Populus*, *Ceratonia*, *Eucalyptus*, *Rosmarinus*, and *Quercus*
P4	Taza	*Ceratonia*, *Silybum*, *Thymus*, *Juniperus*, and *Rosmarinus*
P5	Khénifra	*Eucalyptus*, *Lavandula*, and *Silybum*
P6	Beni Mellal	*Ceratonia*
P7	Salé	*Eucalyptus*, *Euphorbia*, *Juniperus*, and *Quercus*
P8	Témara	*Eucalyptus*, *Quercus*, *Silybum*, *Rosmarinus*, and *Thymus*
P9	Rabat	*Ceratonia*, *Cistus*, *Eucalyptus*, *Thymus*, and *Quercus*
P10	Tiflet	*Ceratonia*, *Eucalyptus*, *Thymus*, *Silybum*, and *Lavandula*
P11	Sidi Kacem	*Citrus*, *Euphorbia*, *Silybum*, *Juniperus*, and *Rosmarinus*
P12	Khnichet	*Citrus*, *Thymus*, *Lavandula*, and *Eucalyptus*
P13	Moulay Bousselham	*Vaccinium*
P14	Jerada	*Citrus*, *Euphorbia*, *Thymus*, and *Lavandula*
P15	Oujda	*Citrus*, *Rosmarinus*, *Silybum*, and *Juniperus*
P16	Ben Slimane	*Eucalyptus* and *Quercus*
P17	Oualidia	*Eucalyptus*
P18	Errachidia	*Euphorbia*, *Eucalyptus*, and *Juniperus*
P19	Sidi Ifni	*Argania* and *Euphorbia*
P20	Tan-Tan	*Euphorbia*

**Table 2:** Physicochemical characterization of the analyzed propolis samples.

Propolis	Wax (%)	Resin (%)	Balsam (%)	Moisture (%)	Ash (%)	pH
P1	61.76 ± 2.09^c^	29.56 ± 0.12^f^	0.84 ± 0.01^j^	1.05 ± 0.09^m^	3.03 ± 0.03^e^	4.7 ± 0.2^a^
P2	29.33 ± 1.09^g^	47.33 ± 0.13^c^	0.67 ± 0.01^kl^	1.79 ± 0.01^j^	4.16 ± 0.01^c^	5.2 ± 0.12^a^
P3	21.31 ± 0.12^h^	58.01 ± 0.09^a^	1.09 ± 0.01^g^	1.02 ± 0.01^m^	5.01 ± 0.01^a^	5.3 ± 0.12^a^
P4	53.64 ± 2.01^d^	27.32 ± 0.09^f^	1.31 ± 0.01^f^	2.32 ± 0.01^e^	2.1 ± 0.01^h^	4.2 ± 0.3^ab^
P5	61.34 ± 3.02^c^	28.82 ± 0.01^f^	0.97 ± 0.01^gh^	2.03 ± 0.06^g^	1.6 ± 0.02^k^	4.5 ± 0.21^ab^
P6	68.08 ± 2.1^b^	18.06 ± 0.02^j^	2.12 ± 0.02^a^	1.96 ± 0.02^h^	2.15 ± 0.01^h^	4.1 ± 0.11^ab^
P7	23.25 ± 0.09^h^	56.21 ± 1.02^b^	1.78 ± 0.01^d^	1.32 ± 0.01^k^	4.52 ± 0.01^b^	5.3 ± 0.2^a^
P8	32.18 ± 0.08^g^	45.7 ± 0.19^cd^	1.89 ± 0.01^c^	1.76 ± 0.01^j^	1.09 ± 0.01^m^	5 ± 0.11^a^
P9	52.84 ± 1.02^d^	42.14 ± 1.09^e^	0.65 ± 0.01^kl^	2.87 ± 0.02^c^	3.39 ± 0.01^d^	5.2 ± 0.2^a^
P10	48.22 ± 1.92^e^	39.3 ± 0.09^e^	1.04 ± 0.01^g^	2.05 ± 0.02^g^	1.49 ± 0.01^j^	4.2 ± 0.21^ab^
P11	47.58 ± 1.23^e^	29.54 ± 0.08^f^	1.67 ± 0.02^e^	2.11 ± 0.01^f^	4.18 ± 0.08^c^	4 ± 0.22^abc^
P12	26.09 ± 1.08^h^	41.06 ± 1.08^e^	0.27 ± 0.01^o^	2.14 ± 0.01^f^	2.04 ± 0.02^hi^	5.1 ± 0.32^a^
P13	46.54 ± 1.03^e^	30.46 ± 0.09^f^	0.86 ± 0.01^j^	2.32 ± 0.02^e^	2.06 ± 0.01^h^	4.8 ± 0.21^a^
P14	30.42 ± 1.02^g^	49.3 ± 0.18^c^	0.56 ± 0.01^n^	2.11 ± 0.02^f^	2.53 ± 0.01^g^	5 ± 0.22^a^
P15	53.23 ± 1.12^d^	22.19 ± 0.19^i^	0.29 ± 0.01^o^	3.01 ± 0.01^b^	2.89 ± 0.01^f^	4.2 ± 0.11
P16	37.38 ± 0.92^f^	48.06 ± 0.14^c^	1.98 ± 0.01^b^	1.87 ± 0.02^i^	3.02 ± 0.02^e^	5.3 ± 0.12^a^
P17	37.18 ± 1.09^f^	23.42 ± 0.12^i^	0.76 ± 0.01^k^	1.12 ± 0.01^l^	4.13 ± 0.01^c^	4 ± 0.11^abc^
P18	60.50 ± 2.01^c^	24.20 ± 0.09^i^	0.96 ± 0.01^gh^	2.45 ± 0.01^d^	1.45 ± 0.02^j^	4.2 ± 0.21^ab^
P19	48.02 ± 1.02^e^	28.08 ± 0.12^f^	0.94 ± 0.02^gh^	2.32 ± 0.02^e^	1.21 ± 0.01^l^	4.4 ± 0.31^ab^
P20	70.12 ± 2.01^a^	17.42 ± 0.09^j^	0.74 ± 0.01^k^	3.65 ± 0.01^a^	0.72 ± 0.02^n^	3.7 ± 0.2^d^

*Note*. Values in the same column followed by the same letter are not significantly different (*p* < 0.05) by Tukey's multiple range test.

**Table 3:** Mineral elements of propolis samples.

Propolis	Ca (mg/kg)	Na (mg/kg)	K (mg/kg)	Mg (mg/kg)	Fe (mg/kg)	P (mg/kg)	Cu (mg/kg)	Zn (mg/kg)	Cd (mg/kg)	Pb (mg/kg)	Cr (mg/kg)	Co (mg/kg)	Ni (mg/kg)
P1	956 ± 10.12^d^	658 ± 8.11^c^	615 ± 11.1^b^	402 ± 12.01^e^	36 ± 2.31^k^	92 ± 10.1^e^	0.4 ± 0.01^abcd^	4 ± 0.01^e^	ND	ND	ND	ND	ND
P2	840 ± 12.01^f^	131 ± 11.01^i^	497 ± 10.11^d^	432 ± 10.51^c^	36 ± 1.79^k^	141 ± 10.1^c^	1.1 ± 0.02^a^	13 ± 0.07^c^	ND	ND	ND	ND	ND
P3	1325 ± 16.1^a^	690 ± 13.11^b^	190 ± 10.12^l^	578 ± 15.1^a^	72 ± 15.11^e^	43 ± 8.1^l^	0.3 ± 0.01^abcde^	5 ± 0.02^e^	ND	ND	ND	ND	ND
P4	575 ± 14.1^k^	125 ± 10.1^j^	275 ± 12.21^g^	134 ± 10.1^j^	80 ± 13.45^d^	95 ± 16.15^e^	1 ± 0.02^a^	4 ± 0.02^e^	ND	ND	ND	ND	ND
P5	475 ± 21.71^m^	610 ± 22.17^d^	210 ± 11.17^k^	230 ± 12.81^h^	53 ± 12.91^h^	70 ± 15.17^h^	0.3 ± 0.01^abcde^	3 ± 0.01^e^	ND	ND	ND	ND	ND
P6	1170 ± 22.1^b^	145 ± 18.19^h^	580 ± 19.41^c^	412 ± 18.81^d^	160 ± 13.1^c^	230 ± 18.12^a^	0.9 ± 0.01^a^	9 ± 0.01^d^	ND	ND	ND	ND	ND
P7	1035 ± 27.12^n^	115 ± 10.17^k^	250 ± 18.12^j^	950 ± 11.17^b^	45 ± 8.91^i^	91 ± 10.1^ef^	0.9 ± 0.01^a^	5 ± 0.01^e^	ND	ND	ND	ND	ND
P8	435 ± 12.12^c^	762 ± 31.01^a^	259 ± 10.09^i^	124 ± 11.89^k^	56 ± 10.1^g^	90 ± 12.91^ef^	0.6 ± 0.01^abcd^	5 ± 0.02^e^	ND	ND	ND	ND	ND
P9	864 ± 18.19^e^	177 ± 19.1^g^	489 ± 21.78^e^	231 ± 19.81^h^	41 ± 8.21^j^	138 ± 10.23^c^	1 ± 0.02^a^	14 ± 0.01^c^	ND	ND	ND	ND	ND
P10	830 ± 32.21^g^	250 ± 10.43^f^	450 ± 14.14^f^	189 ± 14.01^i^	12 ± 5.1^p^	34 ± 8.18^n^	0.4 ± 0.01^abcd^	2 ± 0.01^e^	ND	ND	ND	ND	ND
P11	794 ± 43.16^h^	479 ± 42.01^e^	705 ± 25.28^a^	346 ± 28.14^g^	56 ± 10.18^g^	24 ± 2.91^o^	0.5 ± 0.01^abcd^	1 ± 0.02^ef^	ND	ND	ND	ND	ND
P12	612 ± 34.14^j^	76 ± 10.12^o^	249 ± 11.18^j^	189 ± 12.91^i^	78 ± 9.1^b^	80 ± 9.11^b^	0.5 ± 0.01^abcd^	34 ± 2.01^b^	ND	ND	ND	ND	ND
P13	664 ± 30.12^i^	85 ± 14.15^n^	264 ± 19.18^h^	356 ± 22.11^f^	88 ± 11.12^a^	64 ± 12.17^b^	0.7 ± 0.01^abc^	38 ± 2.31^a^	ND	0.027 ± 0.02^c^	ND	ND	ND
P14	206 ± 19.17^r^	105 ± 11.18^l^	140 ± 10.12^n^	80 ± 10.11^n^	22 ± 3.01^m^	60 ± 5.19^j^	0.5 ± 0.01^abcd^	3 ± 0.07^e^	ND	ND	ND	ND	ND
P15	195 ± 10.13^s^	97 ± 11.12^m^	119 ± 11.31^p^	78 ± 15.1^n^	19 ± 5.14^n^	66 ± 11.1^i^	0.5 ± 0.01^abcd^	3 ± 0.08^e^	ND	ND	ND	ND	ND
P16	293 ± 11.09^o^	51 ± 10.16^s^	127 ± 12.81^o^	69 ± 16.1^p^	29 ± 4.91^l^	56 ± 9.81^k^	0.6 ± 0.02^abcd^	4 ± 0.07^e^	ND	ND	ND	ND	ND
P17	513 ± 12.13^l^	58 ± 11.12^pqr^	274 ± 23.1^g^	102 ± 19.1^l^	61 ± 5.19^f^	79 ± 8.91^g^	0.3 ± 0.01^abcde^	4 ± 0.02^e^	ND	ND	ND	ND	ND
P18	161 ± 17.11^t^	65 ± 10.17^p^	120 ± 24.15^p^	74 ± 18.21^o^	16 ± 3.91^o^	40 ± 5.67^lm^	0.7 ± 0.01^ab^	1 ± 0.01^ef^	ND	ND	ND	ND	ND
P19	261 ± 22.13^p^	63 ± 11.11^p^	186 ± 19.18^m^	90 ± 17.18^m^	29 ± 4.19^l^	118 ± 13.1^d^	0.1 ± 0.01^abcdef^	2 ± 0.01^e^	ND	ND	ND	ND	ND
P20	210 ± 31.1^q^	61 ± 9.23^pq^	116 ± 21.15^pq^	58 ± 21.1^q^	21 ± 5.1^m^	45 ± 6.19^l^	0.8 ± 0.01^a^	1 ± 0.01^ef^	ND	0.02 ± 0.01^b^	ND	ND	ND

*Note*. Values in the same column followed by the same letter are not significantly different (*p* < 0.05) by Tukey's multiple range test. ND: no detected.

**Table 4:** Total phenolic compounds, flavone and flavonol contents, and antioxidant activities of various propolis samples.

Propolis	Phenolic compounds (mg FAE/g)	Flavones and flavonols (mg QE/g)	TAC (mg EAA/g)	DPPH IC_50_ (mg/mL)	ABTS IC_50_ (mg/mL)	FRA IC_50_ (mg/mL)
P1	15.31 ± 1.42^ghij^	9.2 ± 1.35^g^	47.39 ± 0.26^c^	0.856 ± 0.001^k^	0.764 ± 0.005^j^	0.725 ± 0.016^g^
P2	73.75 ± 0.79^d^	40.90 ± 1.42^c^	66.47 ± 2.52^ab^	0.036 ± 0.018^b^	0.083 ± 0.012^d^	0.067 ± 0.005^b^
P3	149.13 ± 2.12^a^	108.11 ± 0.51^a^	80.82 ± 2.16^ab^	0.021 ± 0.001^b^	0.026 ± 0.0007^c^	0.042 ± 0.001^b^
P4	32.41 ± 1.01^g^	10.01 ± 0.78^g^	48.01 ± 0.51^c^	0.046 ± 0.001^bc^	0.578 ± 0.001^i^	0.1008 ± 0.002^c^
P5	12.47 ± 1.31^ghij^	1.71 ± 1.23^k^	9.69 ± 0.47^cdefgh^	0.98 ± 0.001^m^	0.812 ± 0.002^k^	0.85 ± 0.009^h^
P6	38.19 ± 2.43^f^	9.60 ± 1.29^g^	34.58 ± 0.28^cd^	0.052 ± 0.002^bcd^	0.178 ± 0.002^f^	0.115 ± 0.006^c^
P7	96.74 ± 0.12^b^	46.51 ± 3.08^b^	79.91 ± 2.64^a^	0.024 ± 0.0001^b^	0.033 ± 0.0008^b^	0.047 ± 0.003^b^
P8	73.84 ± 3.64^d^	30.21 ± 1.39^de^	43.66 ± 2.2^c^	0.06 ± 0.004^bcde^	0.039 ± 0.038^c^	0.202 ± 0.004^d^
P9	20.84 ± 0.78^ghi^	5.01 ± 1.31^i^	69.77 ± 2.07^a^	0.165 ± 0.012^g^	0.982 ± 0.052^l^	0.749 ± 0.005^g^
P10	41.04 ± 1.64^f^	2.09 ± 1.19^j^	27.81 ± 1.63^cde^	0.146 ± 0.005^g^	0.27 ± 0.01^g^	0.308 ± 0.017^e^
P11	60.14 ± 0.36^e^	21.50 ± 2.24^f^	25.67 ± 0.3^cde^	0.109 ± 0.002^f^	0.091 ± 0.0015^d^	0.133 ± 0.001^c^
P12	63.94 ± 2.64^e^	22.16 ± 1.25^f^	49.26 ± 0.6^c^	0.238 ± 0.007^h^	0.145 ± 0.02^e^	0.138 ± 0.012^c^
P13	30.45 ± 7.03^g^	4.45 ± 1.26^i^	25.73 ± 0.91^cde^	0.236 ± 0.018^h^	0.183 ± 0.012^f^	0.237 ± 0.005^de^
P14	87.14 ± 1.71^c^	37.83 ± 1.12^d^	76.05 ± 0.89^a^	0.029 ± 0.0006^b^	0.085 ± 0.028^d^	0.113 ± 0.008^c^
P15	23.23 ± 0.88^gh^	8.45 ± 1.24^h^	43.2 ± 0.18^c^	0.684 ± 0.02^j^	0.237 ± 0.017^g^	0.326 ± 0.021^e^
P16	74.44 ± 0.59^d^	34.76 ± 4.46^d^	72.72 ± 0.92^ab^	0.05 ± 0.01^bcd^	0.32 ± 0.078^h^	0.226 ± 0.014^d^
P17	8.65 ± 0.72^ghij^	0.41 ± 0.85^l^	18.71 ± 0.66^cdefg^	1.062 ± 0.001^n^	0.132 ± 0.001^e^	0.491 ± 0.002^f^
P18	28.59 ± 2.23^g^	7.56 ± 1.54^h^	35.48 ± 0.38^cd^	0.525 ± 0.038^i^	0.28 ± 0.036^g^	0.226 ± 0.03^d^
P19	12.49 ± 0.21^ghij^	3.12 ± 0.92^j^	23.91 ± 1.05^cdef^	0.949 ± 0.023^l^	0.305 ± 0.001^g^	0.517 ± 0.025^f^
P20	6.74 ± 1.17^ghijo^	1.19 ± 1.36^k^	6.51 ± 1.8^cdefghi^	1.308 ± 0.018^o^	1.529 ± 0.015^m^	1.512 ± 0.106^i^
BHT				0.021 ± 0.01^a^	—	—
Galic acid				—	0.019 ± 0.001^a^	—
Ascorbic acid				—	—	0.035 ± 0.0009^a^

*Note*. Values in the same column followed by the same letter are not significantly different (*p* < 0.05) by Tukey's multiple range test.

**Table 5:** Pearson correlation coefficients between the compositions and antioxidant activity of propolis samples.

	Phenolics	Flavones & flavonols	TAC	DPPH	ABTS	FRAP	Wax	Resin
Phenolics	1	0.934^∗∗∗^	0.686^∗∗∗^	-0.729^∗∗∗^	-0.594^∗∗^	-0.658^∗∗^	-0.793^∗∗∗^	0.859^∗∗∗^
Flavones & flavonols		1	0.626^∗∗^	-0.673^∗∗^	-0.642^∗∗^	-0.594^∗∗^	-0.736^∗∗∗^	0.803^∗∗∗^
TAC			1	-0.558^∗^	-0.560^∗^	-0.570^∗∗^	-0.612^∗∗∗^	0.762^∗∗∗^
DPPH				1	0.907^∗∗∗^	0.896^∗∗∗^	0.559^∗^	-0.643^∗∗^
ABTS					1	0.907^∗∗∗^	0.727^∗∗∗^	-0.420^∗^
FRAP						1	0.674^∗∗^	-0.478^∗^
Wax							1	-0.839^∗∗∗^
Resin								1

^∗^Correlation is significant at the level *p* < 0.05. ^∗∗^Correlation is significant at the level *p* < 0.01. ^∗∗^Correlation is significant at the level *p* < 0.001.

**Table 6:** Antibacterial activity of propolis samples.

Propolis	*Escherichia coli*			*Staphylococcus aureus*			*Pseudomonas aeruginosa*			*Streptococcus faecalis*		
	DI (mm)	MIC (mg/mL)	MBC (mg/mL)	DI (mm)	MIC (mg/mL)	MBC (mg/mL)	DI (mm)	MI (mg/mL)	MBC (mg/mL)	DI (mm)	MIC (mg/mL)	MBC (mg/mL)
P1	12 ± 0.59^b^	1.12 ± 0.07^c^	1.12 ± 0.01^c^	16.3 ± 1.5^b^	0.56 ± 0.01^e^	0.56 ± 0.01^e^	8.02 ± 0.5^a^	1.12 ± 0.07^b^	>1.12	15 ± 0.56^c^	1.12 ± 0.07^d^	>1.12
P2	17 ± 0.89^e^	0.28 ± 0.02^a^	0.28 ± 0.02^a^	20.6 ± 0.5^d^	0.28 ± 0.01^d^	0.56 ± 0.01^e^	9 ± 0.33^b^	1.12 ± 0.07^b^	>1.12	23 ± 1.12^de^	0.56 ± 0.01^c^	1.12 ± 0.07^c^
P3	19.33 ± 2.51^f^	0.28 ± 0.02^a^	0.28 ± 0.02^a^	32.2 ± 1.1^g^	0.002 ± 0.0001^a^	0.002 ± 0.0001^a^	12.3 ± 0.5^bc^	0.56 ± 0.01^a^	1.12 ± 0.01^a^	31.5 ± 1.1^g^	0.07 ± 0.001^a^	0.14 ± 0.01^a^
P4	14.33 ± 1.02^c^	0.56 ± 0.01^b^	0.56 ± 0.01^b^	18.3 ± 1.5^c^	0.07 ± 0.001^b^	0.14 ± 0.01^c^	9.45 ± 0.3^b^	1.12 ± 0.07^b^	>1.12	19.6 ± 1.1^d^	0.56 ± 0.01^c^	1.12 ± 0.07^c^
P5	11.53 ± 0.89^b^	1.12 ± 0.07^c^	1.12 ± 0.07^c^	13 ± 0.59^a^	0.56 ± 0.01^e^	0.56 ± 0.01^e^	10 ± 0.06^bc^	1.12 ± 0.07^b^	1.12^a^	10 ± 0.22^a^	1.12 ± 0.07^d^	>1.12
P6	15.33 ± 1.52^d^	0.56 ± 0.01^b^	0.56 ± 0.01^b^	18.6 ± 0.5^c^	0.07 ± 0.001^b^	0.07 ± 0.001^b^	9 ± 0.56^b^	0.56 ± 0.01^a^	>1.12	19.6 ± 0.5^d^	0.56 ± 0.01^c^	0.56 ± 0.01^b^
P7	18.33 ± 0.57^f^	0.28 ± 0.02^a^	0.28 ± 0.02^a^	27 ± 2.21^f^	0.002 ± 0.0001^a^	0.004 ± 0.0001^a^	10.3 ± 0.6^d^	0.56 ± 0.01^a^	1.12^a^	25 ± 0.33^f^	0.28 ± 0.01^b^	0.56 ± 0.01^b^
P8	16.54 ± 1.18^e^	0.56 ± 0.01^b^	0.56 ± 0.01^b^	19.3 ± 0.5^c^	0.14 ± 0.001^c^	0.28 ± 0.01^d^	10 ± 0.56^bc^	>1.12	>1.12	22 ± 0.56^de^	0.56 ± 0.01^c^	0.56 ± 0.01^b^
P9	12.33 ± 1.01^b^	1.12 ± 0.07^c^	1.12 ± 0.07^c^	15.6 ± 1.1^b^	0.56 ± 0.002^e^	0.56 ± 0.01^e^	9.66 ± 0.6^b^	>1.12	>1.12	12 ± 0.56^b^	1.12 ± 0.07^d^	>1.12
P10	15.22 ± 0.13^d^	0.28 ± 0.02^a^	0.28 ± 0.02^a^	19.1 ± 0.5^c^	0.28 ± 0.001^d^	0.28 ± 0.01^d^	8.26 ± 0.3^a^	>1.12	>1.12	20 ± 0.33^d^	0.56 ± 0.01^c^	0.56 ± 0.01^b^
P11	16.23 ± 1.21^e^	0.56 ± 0.01^b^	0.56 ± 0.01^b^	19 ± 0.29^c^	0.28 ± 0.001^d^	0.28 ± 0.01^d^	10 ± 0.56^bc^	>1.12	>1.12	20.3 ± 0.3^d^	0.56 ± 0.01^c^	1.12 ± 0.07^c^
P12	16.23 ± 1.34^e^	0.56 ± 0.01^b^	0.56 ± 0.01^b^	19 ± 1.73^c^	0.28 ± 0.001^d^	0.56 ± 0.01^e^	9.66 ± 0.2^b^	0.56 ± 0.01^a^	>1.12	20.6 ± 0.5^d^	0.56 ± 0.01^c^	0.56 ± 0.01^b^
P13	14.33 ± 1.33^c^	0.56 ± 0.01^b^	0.56 ± 0.01^b^	18.6 ± 1.1^c^	0.28 ± 0.001^d^	0.28 ± 0.01^d^	8.12 ± 0.6^a^	>1.12	>1.12	19.3 ± 1.1^d^	0.56 ± 0.01^c^	1.12 ± 0.07^c^
P14	17.33 ± 0.57^e^	0.28 ± 0.02^a^	0.28 ± 0.02^a^	22.3 ± 0.5^e^	0.07 ± 0.002^b^	0.07 ± 0.001^b^	9.89 ± 0.1^b^	>1.12	>1.12	20 ± 1.12^d^	0.56 ± 0.01^c^	0.56 ± 0.01^b^
P15	13.33 ± 1.57^c^	1.12 ± 0.07^c^	1.12 ± 0.07^c^	18 ± 1.12^c^	0.28 ± 0.001^d^	0.28 ± 0.01^d^	10.1 ± 1.1^bc^	>1.12	>1.12	16 ± 0.33^c^	0.56 ± 0.01^c^	0.56 ± 0.01^b^
P16	16 ± 0.57^e^	0.56 ± 0.01^b^	0.56 ± 0.01^b^	20.3 ± 1.5^d^	0.14 ± 0.001^c^	0.14 ± 0.01^c^	9 ± 0.66^b^	1.12 ± 0.07^b^	1.12^a^	21 ± 0.56^de^	0.56 ± 0.01^c^	0.56 ± 0.01^b^
P17	11.33 ± 1.15^b^	1.12 ± 0.07^c^	1.12 ± 0.07^c^	15 ± 1.02^a^	1.12 ± 0.07^f^	>1.12	10.3 ± 0.5^bc^	>1.12	>1.12	10 ± 0.12^a^	1.12 ± 0.07^d^	>1.12
P18	13.31 ± 1.12^c^	1.12 ± 0.07^c^	1.12 ± 0.07^c^	18.3 ± 0.6^c^	0.56 ± 0.07^e^	0.56 ± 0.01^e^	9.23 ± 0.3^b^	>1.12	>1.12	18.3 ± 1.5^d^	0.56 ± 0.01^c^	0.56 ± 0.01^b^
P19	12.33 ± 0.57^b^	1.12 ± 0.07^c^	1.12 ± 0.01^c^	12.3 ± 1.5^b^	0.56 ± 0.07^e^	0.56 ± 0.01^e^	10 ± 0.12^bc^	1.12 ± 0.07^b^	>1.12	16 ± 1.02^c^	1.12 ± 0.01^d^	>1.12
P20	8.12 ± 1.09^a^	>1.12	>1.12	—	>1.12	>1.12	—	>1.12	>1.12	—	>1.12	>1.12
Eth70%	—	—	—	—	—	—	—	—	—	—	—	—

*Note*. Values in the same column followed by the same letter are not significantly different (*p* < 0.05) by Tukey's multiple range test.

## Data Availability

The data used to support the findings of this study are available from the corresponding author upon request.
